# Life Is Simple—Biologic Complexity Is an Epiphenomenon

**DOI:** 10.3390/biology5020017

**Published:** 2016-04-27

**Authors:** John S. Torday

**Affiliations:** Evolutionary Medicine Program, University of California, Los Angeles, CA 90095, USA; jtorday@ucla.edu; Tel.: +1-310-222-8186; Fax: +1-310-222-3887

**Keywords:** evolution, life cycle, First Principles of Physiology, unicell, negentropy, chemiosmosis, homeostasis, exaptation

## Abstract

Life originated from unicellular organisms by circumventing the Second Law of Thermodynamics using the First Principles of Physiology, namely negentropy, chemiosmosis and homeostatic regulation of calcium and lipids. It is hypothesized that multicellular organisms are merely contrivances or tools, used by unicellular organisms as agents for the acquisition of epigenetic inheritance. The First Principles of Physiology, which initially evolved in unicellular organisms are the exapted constraints that maintain, sustain and perpetuate that process. To ensure fidelity to this mechanism, we must return to the first principles of the unicellular state as the determinants of the primary level of selection pressure during the life cycle. The power of this approach is reflected by examples of its predictive value. This perspective on life is a “game changer”, mechanistically rendering transparent many dogmas, teleologies and tautologies that constrain the current descriptive view of Biology.

## 1. Evolution from Unicellular to Multicellular

Evolution is an active process for sustaining, perpetuating and adapting life to its environment. Life on Earth originated with the formation of protocells [1], generating bioenergy through chemiosmosis [2] to maintain the intracellular *milieu interieur,* its energetics far from equilibrium, thus circumventing the Second Law of Thermodynamics. About 500 million years ago, unicellular organisms began cooperating metabolically to form multicellular organisms [3], retaining the full multicellular “toolkit” within the unicellular state [4]. That process was fostered by the formation and release of carbon dioxide and oxygen into the nascent atmosphere by Cyanobacteria [5]. The rising oxygen levels fostered the synthesis of cholesterol, which Konrad Bloch, who discovered the cholesterol synthetic pathway, described as a molecular fossil [6]. The introduction of cholesterol into the cell membranes of primitive eukaryotes allowed for the thinning of the phospholipid bilayer, facilitating oxygenation, metabolism and locomotion. These are the fundamental characteristics of vertebrate evolution [7]. Subsequently, cell membrane cholesterol also coalesced to form lipid rafts as the infrastructure for cell surface receptors, providing the basis for cell-cell signaling [8]. Ultimately, cholesterol provided the substrate for steroid hormones and Vitamin D, which are major constituents of the vertebrate endocrine system [9].

We observe these very same interrelationships each time the organism goes through its life cycle, beginning with germline cells producing the zygote, subsequently forming the embryo and fetus through cell-cell interactions mediated by growth factor-growth factor receptor signaling [10]. As a result, the unicellular state remains the primary principle and mechanism of selection pressure, maintaining the equipoise that was established at the very inception of life [11], the multicellular state actually being an epiphenomenon [12].

## 2. The First Principles of Life

The unicellular state is autonomous, able to maintain homeostasis to metabolize, grow, reproduce and evolve [12]. It had its origins in the primitive oceans, which were formed by asteroids composed of frozen water and polycyclic hydrocarbons striking the surface of the Earth freely because the oxygen-less atmosphere could not incinerate them upon entry [1]. The lipids spontaneously formed micelles, semipermeable membrane spheres, when agitated in water due to the effect of the moon on the tides. These structures gained the capacity to regulate calcium using calcium channels [13], entraining calcium ions that were being leached from the ocean floor. The progressive increase in calcium levels was due to the rise in atmospheric carbon dioxide forming carbonic acid in seawater [14]. Later in the emergence of life on Earth, during the Phanerozoic Eon physiologic stress due to the rising, fluctuating levels of oxygen in the atmosphere [15] caused release of calcium stores from the Endoplasmic Reticulum [16], threatening life’s existence due to calcium’s toxic effects on lipids, nucleotides and proteins [13]. In response, such primitive unicellular organisms either acquired or formed Peroxisomes [16], which evolved to neutralize the deleterious effects of leaked calcium using lipids as buffers. The subsequent formation of the nuclear envelope and the endosymbiosis of mitochondria furthered the evolution of unicellular organisms [17].

## 3. The Fractal Nature of Life

The proto-cell possessed all of the qualities necessary for life—the cell membrane, bioenergetics, homeostasis, and reproduction by binary fission. Its primary function was to glean new information from the environment, internalizing it and faithfully communicating the newly acquired knowledge to subsequent generations. Such unicellular organisms dominated the Earth for approximately four billion years [18], ultimately generating organic material in large enough amounts to affect the evolution of the flora and fauna, exapting the self-organizing, self-referential first principles. As a result, the cell remains the fundament of life, not unlike the atom, remaining the smallest functional unit [12].

## 4. What Does Epigenetic Inheritance Tell Us about the Life Cycle

**Figure 1:**
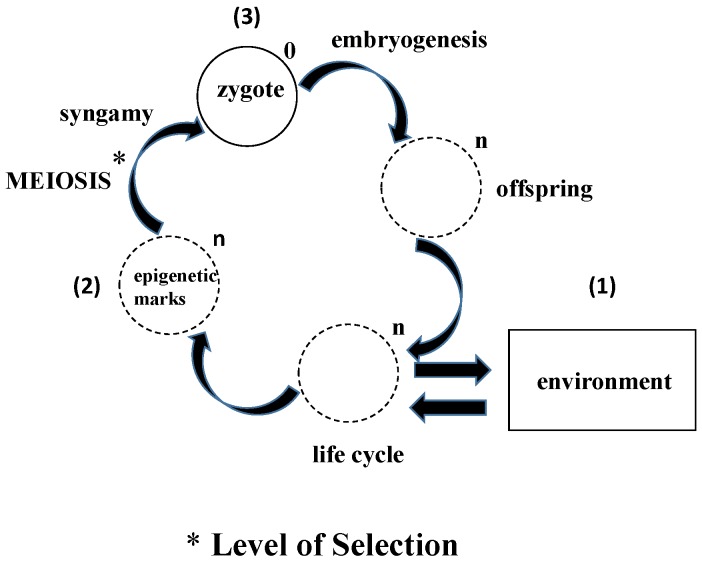
Epigenetic evolution: In contrast to the conventional way in which evolution is seen from the perspective of the adults to the offspring, this schematic portrays the process as Lamarckian evolution from the perspective of the germline cells interacting with the environment. Over evolutionary time (symbolized by the genomic multiplier “n”, reduced back to “0”, based on First Principles of Physiology in the zygote), (**1**) the epigenetic marks accumulated from the environment are (**2**) processed during meiosis, determining which “marks” are assimilated or rejected; (**3**) The processing of the zygote to form the offspring during embryogenesis further determines whether epigenetic marks are incorporated into the genome or not.

The life cycle is dogmatically thought of as the prescribed stages that the organism goes through to ultimately reproduce, completing the biologic loop. Yet epigenetic inheritance bypasses the parents, directly affecting the offspring both genetically and phenotypically [19]. This would suggest that the primary principle and mechanism of selection is actually the unicellular state, and that some of the epigenetic marks acquired during the life cycle are ultimately retained in the gene pool by the germline cells. There are precedents for the primacy of the unicellular state, experiments demonstrating the feasibility of both parthenogenesis [20] and clonal reproduction [21]. Moreover, the direct effects of the environment on the organism via the germline cells are functional homologs of the mechanism by which unicellular life originated as a self-organizing, self-referential process (Figure 1). This mechanistic perspective is one hundred and eighty degrees out of phase with the conventional descriptive view of Biology.

## 5. We Are All Spandrels

In their classic paper “The Spandrels of San Marco and the Panglossian Paradigm: A critique of the adaptationist programme”, Gould and Lewontin [22] attempted to explain that many evolutionary changes in Nature were adaptive, but that there were also biologic changes that were merely phenotypic space fillers, like the spandrels that filled in the gaps in the dome of the Basilica San Marco. That perspective was based on the descriptive view of life, whereas when seen from the cellular-molecular mechanistic vantage point, everything is interconnected through cellular-molecular communications. This basic difference in the interpretation of “spandrels” mechanistically instead of descriptively provides a causal, logical understanding of biology. From the very origins of life, formed by polycyclic hydrocarbons generating micelles, semi-permeable lipid spheres allowed for chemiosmosis generating bioenergy, the homeostatic control of calcium by lipids fostering the biota. All of life is fractals formed from that fundament. Seen from its consequences, it would appear that there are causally- and non-causally-related life forms in Nature, but from an ontologic and epistemologic approach, based on ontogeny and phylogeny, all of Biology can be seen as a continuum.

## 6. What the Unicellular Level of Selection Offers that Is Unavailable to the Top-down Approach

### 6.1. Re-Calibration of Biology and Its Terminology

The conventional perspective on life as having evolved from unicellular to multicellular offers an opportunity to understand the complexity we see in the end-results, but it also masks many aspects of the process such as Lamarckian inheritance, the life cycle, reproduction, death, meiosis, embryogenesis, pleiotropy, heterochrony, and physiology itself as tautologic, teleologic dogma [23].

The realization of this systematic error has largely come from the rediscovery of Lamarckian inheritance, first posited as “The Inheritance of Acquired Characteristics” by Lamarck back in the Eighteenth Century. At the time, he was unable to provide scientific evidence for his theory of the direct effect of the environment on the organism. However, with the advent of molecular methods, we now have evidence that such a mechanism does exist, and probably accounts for many seemingly inexplicable aspects of evolution—natural selection, descent with modification, survival of the fittest, punctuated equilibrium, *etc.* The scientific bottleneck in understanding Lamarckian environmental inheritance was the assumption that the epigenetic marks acquired during the life cycle were eliminated from the germline cells during meiosis. We now know that such epigenetic marks are retained selectively during meiosis [24], and that the process of embryogenesis is a further determinant of which marks are kept or lost during development [25]. Beyond intrauterine developmental mechanisms, it is distinctly possible that the stages of the life cycle themselves are additional determinants of where and when epigenetic marks are acquired. There are specific scientific data that are consistent with this otherwise heretical idea. First, there is evidence that the genes that determine endocrine hormone biosynthesis are affected by epigenetic marks [26], suggesting that active interactions between the individual and its environment affect the timing and degree to which the hormonally-regulated stages of life are expressed; that, in turn, dictates environmental exposures that affect epigenetic inheritance patterns. For example, the food restriction model for the Metabolic Syndrome [27]—obesity, hypertension, and diabetes—also causes precocious puberty. The earlier onset of sexual maturity in the context of low food abundance suggests that the selection advantage is for accelerating the transition of the offspring to the next hopefully more food abundant environment. The disruption of the allostatic mechanisms controlling body habitus (obesity), blood pressure (hypertension) and control of blood sugar (type II diabetes) are secondary consequences of maternal food restriction, as is senescence during the normal life cycle, all of which are consequences of the otherwise adaptive acceleration of the life cycle [28].

Further evidence for the functional interrelationship between nutrient availability and the reproductive strategy comes from the slime mold Dictyostelium. When deprived of nutrients, the free-swimming form of Dictyostelium reverts to the sessile colonial phenotype. The determinant of this phenotypic change is the Target of Rapamycin (TOR) gene [29], a regulatory protein kinase that has also been implicated in the food restriction model of metabolic syndrome [30]; the TOR gene is involved in vertebrate physiologic regulation of ions, nutrients, physical forces, oxygen, and controls cell growth.

### 6.2. Deep Understanding of Physiology

The molecular era for understanding Physiology was launched by Konrad Bloch’s discovery of the biosynthetic pathway for cholesterol [6]. He had the insight to hypothesize that cholesterol was a molecular fossil, requiring eleven atoms of oxygen to synthesize one molecule of cholesterol. Oxygen levels began rising and falling in the atmosphere about 500 million years ago [15], being produced initially by cyanobacteria. The notion that there was a fossil record based on molecular data, not just based on mineralized bone, was a breakthrough in our conception of life’s origins and makings. Zuckerkandl and Pauling [31] formulated “molecular clocks” that allowed them to identify the phylogenetic origins of organisms, but that was still just a further reduction of the descriptive approach to Biology initiated by Linnaeus [32]. It is only recently that we have applied molecular principles to ontogeny and phylogeny in order to determine the unicellular origins of complex physiologic structures and functions [12]. In tracing the unicellular membrane origins of the lung by exploiting the role of cholesterol in adaptation to gas exchange [12], there was the realization that the underlying functional genomic characteristics were also common to other tissues and organs [12], allowing for a prograde re-conceptualization of how and why complex physiologic traits evolved, such as the kidney, gut, liver, brain and skin [12].

### 6.3. A Brief “History” of Lung Surfactant

As alluded to above, the insertion of cholesterol into the phospholipid bilayer of unicellular organisms facilitated vertebrate evolution, enhancing metabolism, respiration and locomotion, the three basic traits for vertebrate evolution [7]. Cholesterol is the most primitive of lung surfactants, likely having evolved from its lubricating effect in the swim bladder of boney fish [33]. This exaptation of cholesterol for gas exchange from unicellular organisms to mammals and birds has been recounted by Orgeig and Daniels [34]. The interrelationship between cell-cell communication, lung morphogenesis, physiology and the evolution of the surfactant system has been elucidated by Torday and Rehan [35,36,37,38].

### 6.4. Interrelationships between Human Physiology and that of Other Organisms

Such deep knowledge of complex Physiology having its origins in the First Principles of Physiology in the unicellular state also allows for a functional understanding of comparative physiology that transcends simple analogy, opening up to a true understanding of homology of structure and function [39,40]. For example, it had long been assumed that the lung evolved from the gills of fish, given that the latter were the phylogenetic analog for gas exchange, and because there were many anatomical and other inconsistencies between the lung and swim bladder [39,40]. However, once it was established that the molecular biology of swim bladder development was virtually the same as that of the lung [41], as well as functional genomic homologies, this controversy was resolved. It was what the Nobelist Francois Jacob described as “tinkering” [42]—in the swim bladder, gas exchange allows fish to adapt to buoyancy, optimizing metabolism in the process, aided by the most primitive surfactant, cholesterol, produced by the gas gland epithelial cells [41]; on the other hand, the lung utilizes oxygen directly for metabolic advantage, also facilitated by lung surfactant, optimizing the surface area-to-blood volume ratio as a result of tinkering. Both organs express Parathyroid Hormone-related Protein (PTHrP), which is necessary for alveolar formation in land vertebrates [43].

PTHrP expression in the kidney glomerulus likely facilitated the evolution of the fish kidney glomus, a simple kidney capillary system, into the complex glomerulus in land vertebrates since PTHrP is angiogenic, fostering capillary formation for greater homeostatic control of fluid and electrolytes [44].

PTHrP expression is critically important for the structure and function of many land vertebrate physiologic traits—lung, kidney, bone, and skin. It is expressed in all epithelial cells, and its receptor resides on the surface of adepithelial mesodermal fibroblasts [45]. The PTHrP Receptor duplicated during the water–land transition [46], mediating terrestrial traits by effectively amplifying the PTHrP signal in a tissue- and organ-specific manner. This was likely due to the shear stress generating Radical Oxygen Species in the microvasculature of these specific organs since they were the ones most stressed by breaching land [47]. Another gene that was duplicated during this existential transition was the βAdrenergic Receptor (βAR) [48], due to the constraint of lung evolution by βAR regulation of both the pulmonary and systemic blood pressures [49]. Positive selection for the increased expression of βARs in the lung allowed for its independent regulation of vascular dilatation for efficient gas exchange [50]. Moreover, because of its central role in heart development [51], the amplification of the βAR could also have facilitated the evolution of the heart ventricles from one in worms, to two in fish, three in birds and four in mammals. To corroborate the relevant interrelationship between βARs and heart evolution, deletion of the βAR in embryonic mice results in the development of only one ventricle [51], which is homologous with the earlier phylogenetic heart of a worm.

### 6.5. Predictive Value of the Comparative Cellular–Molecular Approach

**Figure 2:**
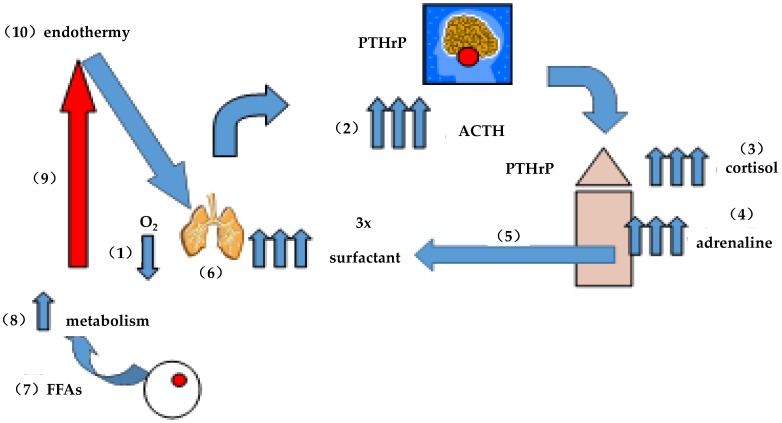
On the evolution of endothermy: (**1**) intermittent hypoxia caused physiologic stress; (**2**) stimulating pituitary ACTH; (**3**) ACTH stimulated corticoid production; (**4**) stimulating adrenaline production; (**5**) adrenaline stimulated surfactant secretion by the alveoli; (**6**) increasing alveolar distension/surface, area for gas exchange alleviating the hypoxic stress acutely; (**7**) adrenaline also stimulates fatty acid (FFA) secretion by adipocytes; (**8**) increasing metabolism; (**9**) and therefore body temperature; and (**10**) ultimately giving rise to endothermy. Surfactant phospholipid is three times more surface active at 37 °C than it is at 25 °C, accommodating lung evolution.

One can easily argue against these physiologic adaptations being causal since there is no physical fossilized evidence for this sequence of events, though the functional interrelationships are consistent with their contemporary roles in ontogenetically forming and phylogenetically maintaining homeostasis *a posteriori* [52]. There is also an *a priori* scenario for the subsequent evolution of these integrated physiologic traits that is internally consistent with their ontogeny and phylogeny through the advent of endothermy/homeothermy [38]. Since a non-teleologic explanation for the evolution of endothermy/homeothermy has not previously been formulated [53], by exploiting the above-mentioned gene duplications, a hypothetical mechanism is proposed that entails exaptation of pre-existing physiologic traits that may conditionally have given rise to endothermy/homeothermy. Briefly [38], during vertebrate adaptation to land, periodic episodes of hypoxia would have occurred as the lung evolved (Figure 2). Hypoxia would have caused physiologic stress, stimulating the pituitary-adrenal axis, increasing adrenal corticoid production, and stimulating adrenaline secretion by the adrenal medulla [54]. Elevated levels of circulating adrenaline would have relieved the hypoxic constraint on the lung by stimulating surfactant secretion by the alveoli [55], increasing their distensibility, and thus their surface area for oxygenation. In tandem, adrenaline would also have stimulated fatty acid secretion by peripheral fat pads [56], stimulating metabolism [57] and body temperature [58]. The selection advantage for the increased, stable body temperature would have given rise to endothermy [59] since being worm-blooded is much more energetically efficient than being cold-blooded, endothermic homeotherms requiring only one form of any given metabolic enzyme, whereas poikilotherms have to express several isotypes of the same enzyme to function effectively at varying body temperatures [60].

The increase in body temperature would have interacted synergistically with the evolved mammalian lung surfactant phospholipids, primarily composed of saturated phosphatidylcholine that functions 300% more actively to reduce surface tension at 37 °C than at 25 °C. This effect is due to the elevated phase transition temperature of saturated phosphatidylcholine (41 °C), the temperature at which the alveolar lung surfactant film collapses, no longer able to reduce surface tension [61]. The selection pressure for the co-evolution of saturated phosphatidylcholine production by the alveoli and endothermy/homeothermy may have been due to the pleiotropic effects of adrenaline, stimulating both surfactant secretion by the alveoli, and coordinately increasing the unsaturated fatty acid composition of peripheral cell membranes, thereby increasing oxygen uptake by increasing cell membrane fluidity [62]. The phylogenetic increase in the percentage of saturated phosphatidylcholine in lung surfactant [34] is indicative of the constitutive change in adaptation to endothermy/homeothermy.

These fundamental changes in lipid composition in service to metabolism are exaptations of the facilitation of oxygenation by cholesterol in unicellular eukaryotes [35,38]. Considering the severe conditions generated by Romer’s Gap [63], during which vertebrates were virtually wiped off the face of the Earth, it is not surprising that such deep homologies were reprised during this critical phase of vertebrate evolution.

## 7. Why Did Receptor Genes in Particular Duplicate

It is notable that the three gene duplications that fostered the vertebrate adaptation to land were all for receptor expression—the PTHrP, βAdrenergic and Glucocorticoid Receptors. Elsewhere [59], it has been suggested that these duplications occurred as a result of the physiologic stress generated by the water–land transition causing shear stress within the microcirculatory beds of those specific tissues most physically affected—the lung, kidney and skin. The generation of Radical Oxygen Species [47] could have caused mutations and duplications within those specific niches, governed by evolved structural and functional traits. The receptors are mediators of the ligands that specifically bind to them, triggering second messengers that primarily affect homeostasis, and secondarily affect morphogenesis. Duplication of the ligand would not have been as effective as duplication of the receptor, which naturally amplifies the ligand signal, by definition, functionally and structurally compensating for the trait being constrained. Over and above that feature, duplication of the receptor would potentially have affected the down-stream signaling mechanisms and phenotypes involved, begging the question as to how this is accomplished evolutionarily? It should be borne in mind that all evolved vertebrate physiologic traits had their origins in the unicellular state [12], and that exapted traits must behave in accordance with the First Principles of Physiology [35,38,59]. It is the First Principles of negentropy, chemiosmosis and homeostasis as applied to reproduction that accomplishes this, both during meiosis to rule in or rule out epigenetic marks discordant with the genome, and during embryogenesis, acting as a “functional template” for any newly acquired genetics or epigenetics.

## 8. The Exception that Proves the Rule

The other gene mutation known to have occurred during the vertebrate adaptation to land was the Goodpasture type IV collagen isomer alpha3 (IV) NC1 [64]. It is more hydrophobic than the other type IV collagens that form physicochemical barriers for epithelial cells [64], preventing water loss across the alveolus and glomerulus, facilitating adaptation for vertebrate evolution during the water–land transition. Like the evolution of the duplicated receptor genes, type IV collagen was constrained by the functional niche in which it existed. But unlike the PTHrP Receptor and the βAR, it was not mechanistically servo-regulated by a complex, evolved “history” of homeostatic regulatory mechanisms. Perhaps that is why the evolution of the Goodpasture isomer resulted in disease in some individuals, albeit a very small subset [65].

Thus, what Francois Jacob described as “tinkering” can now be understood mechanistically as the organism ascribing and adhering to the First Principles of Physiology of the unicellular state. The so-called evolutionary “tinkering” is determined by the constraints of the cellular–molecular context, *i.e.*, it is deterministic, not probabilistic.

## 9. Hibernation as Reverse Evolution of Endothermy/Homeothermy

The causal nature of the interrelationship among physiologic stress, catecholamines and endothermy/homeothermy is substantiated by the obverse effects of hibernation, or torpor, on lung surfactant lipid composition and peripheral cell membrane fatty acid composition. Under such low stress conditions, decreased catecholamine production results in both increased surfactant cholesterol, rendering lung surfactant less surface active, and decreased unsaturated fatty acid content of cell membranes [59], adaptively reducing oxygen uptake.

There is a phylogenetic precedent for lung surfactant facultatively accommodating ambient temperature. For example, in a study by Lau and Keogh [66] it was found that maintaining map turtles at different ambient temperatures adaptively altered the composition of their lung surfactant. Ultimately, the ability to optimize lung alveolar physiology at various environmental ambient temperatures may have been the precursor to endothermy/homeothermy. Experimental evidence for the causal interrelationships among body temperature, surfactant composition and catecholamine regulation of surfactant secretion [59] supports this hypothesis.

Moreover, it is this causal interrelationship between vertebrate land adaptation, the neuroendocrine and respiratory systems, and endothermy/homeothermy that may have been why Weibel and his colleagues [67] found that the lung is physiologically “over-engineered”. In seeking to determine whether biological organisms are structurally evolved to match their functional demand, which they termed symmorphosis, Weibel, *et al.* discovered that this held true for all the internal compartments of the respiratory system (blood, heart, muscle capillaries, and mitochondria) except the lung. They concluded that for some unknown reason the physiologic capacity of the lung exceeds its physical requirements. From a descriptive Darwinian standpoint, that may have been because of the existential role of the lung in land vertebrate evolution, positively selecting for those organisms with optimal gas exchange capacity. However, from a mechanistic standpoint, based on the exaptation of atavistic cellular–molecular traits, it may reflect the underlying positive evolutionary selection pressure for PTHrP/PTHrP Receptor signaling in facilitating the adaptation of multiple organs for life on land [35,38]—those organisms with the most robust PTHrP-PTHrP signaling capacity would have been the most adaptive, hence the seeming “over-engineering” of the lung. Those faculties are also reflected by the essential nature of the lung in complex pathophysiology [35,38].

The cellular accommodation of environmental temperature by lipids is hypothetically an exaptation of the fundamentally enabling effects of cholesterol at the origins of eukaryotic evolution [35]. That this is not merely an association is corroborated by the evolution of the alveolar lipofibroblast in mammals [37]. These adipocyte homologs, located within the alveolar wall contiguous with the alveolar epithelial cells that produce surfactant provide a ready source of lipid substrate for increased surfactant phospholipid production under physiologic demand for oxygen via the stretch-regulated mechanism described above. As further evidence for this hypothetical evolutionary mechanism, when cholesterol synthesis by alveolar type II cells is experimentally eliminated from the developing mouse lung by deleting the *Scap* gene, the lung alveoli effectively compensate for the loss of cholesterol by increasing the number of lipofibroblasts [68], seemingly recapitulating their evolutionary origins. This compensatory mechanism is apparently due to the observed concomitant increase in PPARγ expression by these cells [68], likely due to endoplasmic reticulum stress reprising why peroxisomes evolved in the first place [16]. It is precisely such atavistic traits that can be exploited for the effective diagnosis and treatment of disease, as well as understanding what functionally constitutes “health” [35], which is still defined as the absence of disease, and *vice versa*.

As further evidence in support of the hypothesized role of hypoxia-induced endothermy/homeothermy, there are other significant mammalian-specific changes that occurred during vertebrate evolution that are functionally consistent with this mechanism. First, PTHrP appears in both the mammalian pituitary [69] and adrenal cortex [70], thus amplifying the fight-or-flight mechanism. Furthermore, Richard Wurtman [54] has discovered that the capillary arcades in the mammalian adrenal medulla are phylogenetically more complex, acting to amplify the production of catecholamines under stress conditions, as follows: in response to pituitary ACTH stimulation, glucocorticoids produced in the adrenal cortex pass through the adrenal medulla, where they stimulate the rate-limiting step in catecholamine biosynthesis, Catechol-O-Methyl Transferase [71], enhancing adrenaline production for the stress reaction. This expansion of the medullary microvasculature may itself have been caused by the adrenocortical secretion of PTHrP, since PTHrP is angiogenic [72]. Speculatively, the coordinated effects of PTHrP on the pituitary, adrenal cortex and medulla may have fostered the structural integration of the independent cortical and chromaffin tissues of fish in transition to the amphibian corticomedullary configuration [73].

It is also feasible that this complex cascade of physiologic stress-mediated cellular mechanisms gave rise to the kidney glomerulus, which is largely absent in fish, but is omnipresent in amphibians, reptiles, mammals and birds [74]. PTHrP is the mediator of fluid and electrolyte balance in the glomerulus, being secreted by the podocytes lining this compartment, binding to its receptor on the mesangium, which regulates the flow of fluid and electrolytes entering the kidney tubules [75]; as is the case for the lung alveolar epithelium, the fluid distension of the glomerulus is sensed by the podocyte, which then transduces that signal for fluid and electrolyte balance via PTHrP signaling to the mesangium [76]. Here again is a functional homology between seemingly structurally and functionally disparate tissues and organs based on descriptive biology, representing the pleiotropic distribution of the same cellular–molecular trait for both air breathing and for fluid and electrolyte balance. The latter trait may also have evolved under the influence of increased catecholamine production due to physiologic stress, since epinephrine inhibits loss of water and salt from the kidney [77] in adaptation to land.

In further support of this complex scenario for the evolution of land vertebrate physiology, it has been observed that the genome decreased by about 80%–90% after the Cambrian Extinction [78]. The advent of endothermy may explain this phenomenon because ectotherms require complex enzymatic regulatory mechanisms in order to accommodate variable atmospheric temperatures, whereas the uniform body temperature of endotherms/homeotherms only requires one metabolic isoform to function optimally. Since metabolic genes account for 17% of the human genome [78], representing a fraction of the number of metabolic genes expressed by ectotherms, this reduction in metabolic enzyme heterogeneity would have contributed to the dramatic decrease in post-Cambrian genomic size.

## 10. Predictive Power of the Cellular–Molecular Approach to Evolution

Starting with the perspective of the unicellular stage as the primary principle and mechanism of selection during the life cycle [38], and therefore necessitating the iterative return to it as the adaptive strategy for epigenetic inheritance, the cellular–molecular approach is highly predictive, particularly in comparison to the conventionally dogmatic, descriptive view of biology that we have held for thousands of years. The recognition that the cell membrane is the homolog for all complex physiologic traits [38] forms the basis for understanding the First Principles of Physiology. By focusing on the mechanistic transition from the unicellular state to the multicellular organism during both ontogeny and phylogeny, such seemingly enigmatic properties of life as pleiotropy [79], the stages of the life cycle and the aging process can all be understood as one continuous process in service to evolutionary emergence and contingence.

Perhaps more to the point, regarding the predictive power of the cellular approach to evolution, it has recently been hypothesized that among amniotes the alveolar lung of mammals may have been the earliest adaptation for land life, followed by its simplification in snakes and lizards [80]. There is no mechanistic basis for such speculation, as interesting and provocative as this idea is; in fact, it runs counter to the developmental pattern of the mammalian lung, which begins as simple sacs that become progressively more structurally complex, consistent with the phylogeny of the lung evolving from the swim bladder. It has previously been pointed out that there is a systematic error made in showing associations in evolution without offering a mechanistically causal relationship to environmental factor(s), particularly at the cellular–molecular level in an attempt to determine relationships to other interrelated evolutionary mechanisms [35,36,37,38], given the complex nature of this process. In that spirit, a hypothetical role for physiologic stress in mammalian lung evolution to other amniotes with “simple” lungs has been applied. The simple sac-like lungs of amniotes is associated with a difference in the configuration of the adrenal glands between mammals and other amniotes; it is helpful here to keep in mind that the adrenal gland is composed of a separate cortex and medulla. In mammals, the adrenal cortex is caudad to and structurally-functionally connected with the medulla. The corticoids secreted by the cortex pass down through the medulla, amplifying adrenaline production by stimulating Catechol-O-methyltransferase, the rate-limiting step in adrenaline synthesis. In all other amniotes, the chromaffin cells that synthesize catecholamines are dispersed within the cortical tissue, and the relationship between stress and adrenaline production is not functionally integrated as an evolutionary selection pressure.

Clearly, non-mammalian amniotes evolved a different mechanism to cope with the physiologic stresses of land adaptation, and seemingly as a consequence, their adaptation for breathing as well. The comparators are birds, which have a “stiff”, non-reciprocating lung composed of much larger air sacs [81]. The lungs are affixed to the dorsal wall of the thorax during embryogenesis [81]. Furthermore, air entering the lung flows in only one direction [81], unlike the bellows-like nature of the mammalian lung, indicating a fundamentally different evolutionary strategy for adapting to air breathing in birds. Embryonic alligators also exhibit the attachment of the lung to the chest wall during embryogenesis [82], and in the adult [83] in association with unidirectional air flow, in further support of the speculation that affixing the lung to the chest wall during development is in service to the unidirectional flow of air. This supposition is further substantiated by the fact that birds have blood glucose levels 1.5–2 times higher than mammals [84], suggesting that instead of secreting fatty acids from fat stores in response to adrenaline for metabolic “fuel” on an “as needed” basis via the fight-or-flight physiologic stress mechanism used by mammals, birds are constantly in a “metabolically-on” mode. Moreover, it is noteworthy in the context of metabolic evolution that both birds and humans are bipeds, which may hypothetically have been a consequence of their both being endotherms. Being upright is metabolically costly [85], but by increasing their body temperature in adaptation to land and air, both humans and birds, respectively, have become much more metabolically efficient; as mentioned earlier, cold-blooded organisms require elaborate metabolic control mechanisms to function efficiently at variable ambient temperatures [86], whereas endotherms usually have only one [85]. Bipedalism may have resulted, freeing the forelegs to evolve into wings and hands through common genetic motifs [85].

The hypothesized evolutionary physiologic interrelationship between stress, metabolism and endothermy may underlie the effect of meditation on hypometabolism [86]—it has long been known that Yogis have the capacity to regulate their metabolism at will [87], and formal study of this phenomenon has validated it scientifically [88,89]. Functionally linking to ever-deeper principles of physiologic evolution through meditation and biofeedback may prove to be of wider benefit in healing, both conventional and self-healing alike.

## 11. Fractal Basis for Complex Physiology Reveals Its Unicellular Essence

The (re)iterative motif of physiologic stress acting through calcium/lipid epistatic homeostasis to both generate and repair form and function reveals the fundamental fractal nature of physiology, from unicellular to multicellular organisms. Life begins with a “spark” of calcium both phylogenetically and ontogenetically, signaling to the internal milieu for stasis or change depending upon the nature of the signal. Ontogenetically, when sperm meets egg the first process that is activated is a calcium burst [90], followed thereafter by embryogenesis; phylogenetically, when paramecia encounter nutrients in their environment it is signaled to the organism by a calcium wave [91].

Consider the response of the rat dam to a 50% reduction in nutrient during the second half of gestation [92], giving rise to small-for-gestational age offspring. As a consequence, the offspring experience premature adrenarche, the hormonal trigger for puberty [93]. In other words, the food restriction truncates the offspring’s life cycle by reproducing sooner, offering the opportunity for the next generation to transit to a potentially more food-abundant environment. This is homologous at the cellular–molecular level with the response of the free-swimming amoeboid Dictyostelium to food deprivation—nutrient scarcity activates the *TOR* gene, which signals through down-stream signaling mechanisms to trigger the colonial slime mold phenotype [94]. The *TOR* gene also mediates nutrient control in the rat [95].

## 12. Conclusions

Seen from its superficial appearances, physiologic characteristics are highly complex and “inscrutable” [96,97]. But that analysis of physiology is based on a synchronic “snapshot”, yet it has evolved over eons, largely in response to the ever-changing environment. Starting from the origins of life on Earth as the mixing of “oil and water” deposited by asteroids striking the surface of the Earth, unprotected by an oxidative atmosphere [1], one can trace the arc of physiologic evolution [35,38]. That perspective, combined with what we now know about epigenetic inheritance directly from the environment provides a fresh view of the process emanating from, but never diverging from the original unicellular strategy. This is a testable and refutable hypothesis, which offers answers to what are otherwise dogmatic tautology—why do we go through the life cycle? Why do we return to the unicellular state? Why senescence? The notion that there are First Principles of Physiology [35,38], and that they are knowable and testable provides a whole new vista for Biology, not unlike the realization that the Earth is round, and is not the center of the Solar System.
